# Expression and serum levels of the neural cell adhesion molecule L1-like protein (CHL1) in gastrointestinal stroma tumors (GIST) and its prognostic power

**DOI:** 10.18632/oncotarget.27525

**Published:** 2020-03-31

**Authors:** Karl-Frederick Karstens, Eugen Bellon, Adam Polonski, Gerrit Wolters-Eisfeld, Nathaniel Melling, Matthias Reeh, Jakob R. Izbicki, Michael Tachezy

**Affiliations:** ^1^Department of General, Visceral and Thoracic Surgery, University Medical Center Hamburg Eppendor, Hamburg, Germany

**Keywords:** neural cell adhesion molecule L1-like protein (CHL1), gastrointestinal stroma tumor (GIST), survival, prognosis, serum

## Abstract

Introduction: Diagnosis of gastrointestinal stroma tumors (GIST) is based on the histological evaluation of tissue specimens. Reliable systemic biomarkers are lacking. We investigated the local expression of the neural cell adhesion molecule L1-like protein (CHL1) in GIST and determined whether soluble CHL1 proteoforms could serve as systemic biomarkers.

Material and Methods: Expression of CHL1 was analyzed in primary tumor specimens and metastases. 58 GIST specimens were immunohistochemically stained for CHL1 on a tissue microarray (TMA). Systemic CHL1 levels were measured in sera derived from 102 GIST patients and 91 healthy controls by ELISA. Results were statistically correlated with clinicopathological parameters.

Results: CHL1 expression was detected in GIST specimens. Reduced tissue expression was significantly associated with advanced UICC stages (*p* = 0.036) and unfavorable tumor localization (*p* = 0.001). CHL1 serum levels are significantly elevated in GIST patients (*p* < 0.010). Elevated CHL1 levels were significantly associated with larger tumors (*p* = 0.023), advanced UICC stage (*p* = 0.021), and an increased Fletcher score (*p* = 0.041). Moreover, patients with a higher CHL1 serum levels displayed a significantly shortened recurrence free survival independent of other clinicopathological variables.

Conclusion: Local CHL1 expression and serum CHL1 levels show a reverse prognostic behavior, highlighting the relevance of proteolytic shedding of the molecule. The results of the study indicate a potential role of serum CHL1 as a diagnostic and prognostic marker in GIST.

## INTRODUCTION

Gastrointestinal stromal tumors (GISTs) present a broad clinical spectrum of symptoms due to their various localizations in the gastrointestinal tract and malignant behavior. They are the most common mesenchymal neoplasms in the gastrointestinal tract and are typically identified by expression of tyrosine kinase receptors, c-kit (CD117) and platelet-derived growth factor receptor alpha (PDGFRa) [1–4]. Several prognostic parameters were identified and included in clinical staging such as primary tumor size, nodal and metastatic status, mitotic rate and localization of the tumor [5]. GISTs are often diagnosed incidentally at endoscopic or surgical procedures as well as during the evaluation of patients suffering from unspecific abdominal symptoms or upper gastrointestinal bleeding [6]. Until now, diagnosis and prognosis are based on histopathological examination of biopsy or surgical samples since no serum markers exist. Hence, there is an urgent need for novel prognostic markers and potential therapeutic target molecules to improve diagnosis and treatment strategies.

GISTs originate from the interstitial cells of Cajal located in the nerve plexus of the muscularis in the gut wall [3, 7]. Therefore, several neuronal molecules are expressed in GIST. In other tumors, neuronal adhesion molecules of the L1 family like L1 (CD171), NrCAM and Neurofascin have been extensively investigated [8–13]. However, few is known about is the neural cell adhesion molecule L1-like protein (CHL1), which is another L1 family member. CHL1 is a multidomain type 1 membrane glycoprotein of the immunoglobulin superfamily which has analogous physiologic functions in the development of the neuronal system as L1 [14]. Two isoforms of CHL1 are expressed, of which isoform 2 is characterized by the additional mini-exon 8. The physiological relevance of these isoforms is unknown. CHL1 participates in the nerve cell regeneration und cortical development, proliferation and migration of neurons and acts as a survival factor for motoneurons [15, 16]. Similar to L1, which is detectable in ascites or blood serum of patients in its soluble form, the ectodomain of CHL1 is cleaved by a disintegrin and metalloproteinase 8 (ADAM8) and beta-secretase (BACE1) from the cell surface [17–19]. This ectodomain promotes neurite outgrowth and suppresses neuronal cell death [20]. An increasing number of studies demonstrated a role of CHL1 in cancer growth, invasion and migration for different entities. He et *al.* described a downregulation of CHL1 in breast cancer and an association with lower tumor grading. On the other hand, overexpression seemed to suppress the invasion and proliferation of tumor cells [21]. Manderson et al. reported that compared to healthy ovarian tissue gene expression of CHL1 is elevated in serous epithelial ovarian cancer [22]. Furthermore, CHL1 expression in esophageal and lung cancer is significantly correlated with a favorable outcome [23, 24]. In addition, a functional role as a tumor suppressor in the Akt pathway in esophageal cancer was found [25]. Another recent study also demonstrated a tumor suppressive function of CHL1 in neuroblastoma [26]. In addition, a down-regulation of CHL1 via overexpression of miR-21-5p promotes the propagation and invasion of tumor cells in colon adenocarcinomas [27]. Our group has recently described a significant role of CHL1 expression in non-small-cell-lung cancer and a correlation with overall survival of the patients [24].

Hence, the neuronal adhesion molecule CHL1 might also play a role in the genesis of GIST. This study investigates the expression of CHL1 and evaluates its association with clinical and pathological aspects in GISTs. Furthermore, we investigated the potential of the shed CHL1 as a peripheral tumor marker in sera of GIST patients.

## RESULTS

### CHL1 is expressed in GIST and the majority is proteolytically cleaved

**Figure 1:**
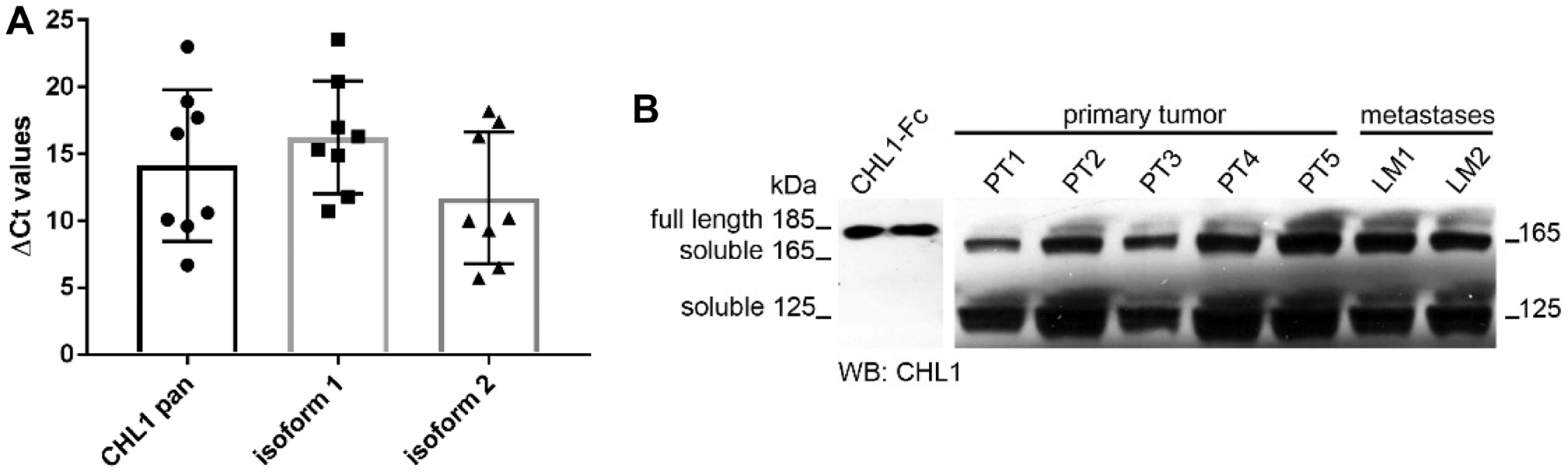
CHL1 expression analysis in GIST. (**A**) Relative mRNA expression levels of pan CHL1, and isoforms 1 and 2 in primary GIST, (**B**) Western blot analyses of CHL1 protein expression in primary tumors and metastases.

To investigate the expression of CHL1 in GIST, we used qPCR to determine the relative mRNA expression levels of CHL1 and its isoforms 1 and 2 in eight GIST primary tumors (PT). In all examined samples CHL1 transcripts were detected. Furthermore, it was shown that, with one exception, both CHL1 isoforms are expressed in GIST. (Figure 1A). On protein level, we further confirmed CHL1 expression in five primary tumors, and two distant metastases. All samples displayed a distinct CHL1 expression in Western blot analysis. Interestingly, the soluble proteolytic CHL1 fragments with the molecular weights of 165 kDa and 125 kDa were the most prevalent, while full length CHL1 with the molecular weights of 185 kDa was only detectable in an extenuated form (Figure 1B).

### Reduced local CHL1 expression correlates with advanced tumor stages

**Figure 2:**
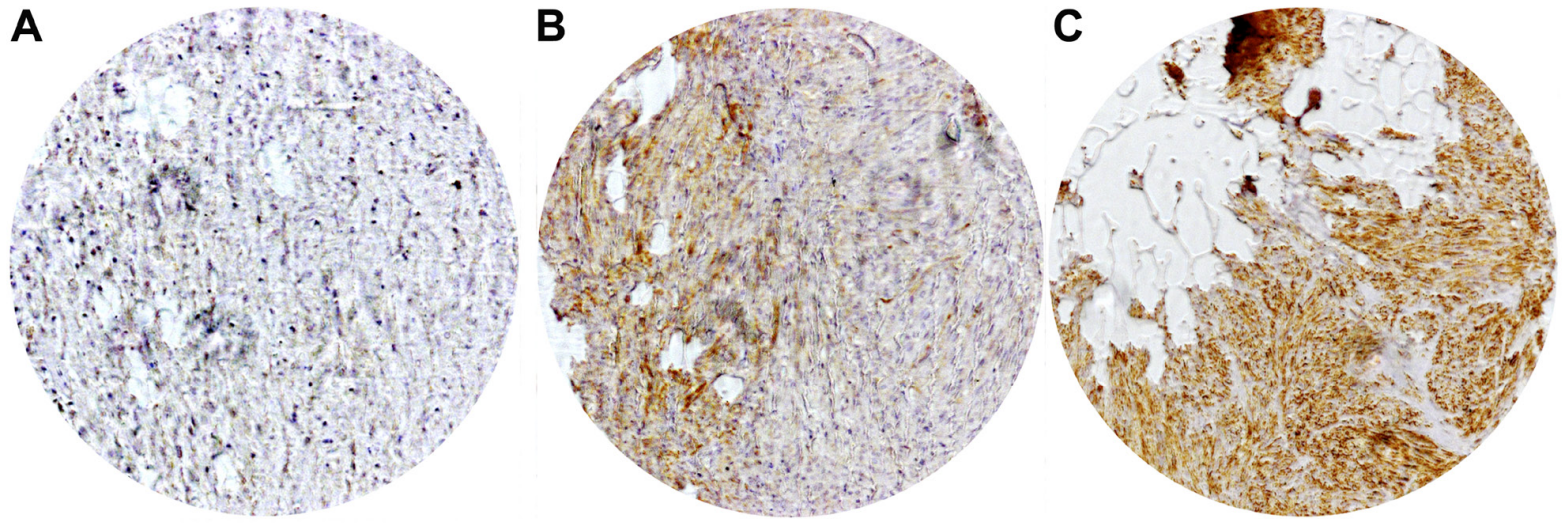
Representative immunohistochemical staining of local CHL1 expressions in GIST. (**A**) No expression (**B**) Low expression (**C**) High expression.

**Figure 3:**
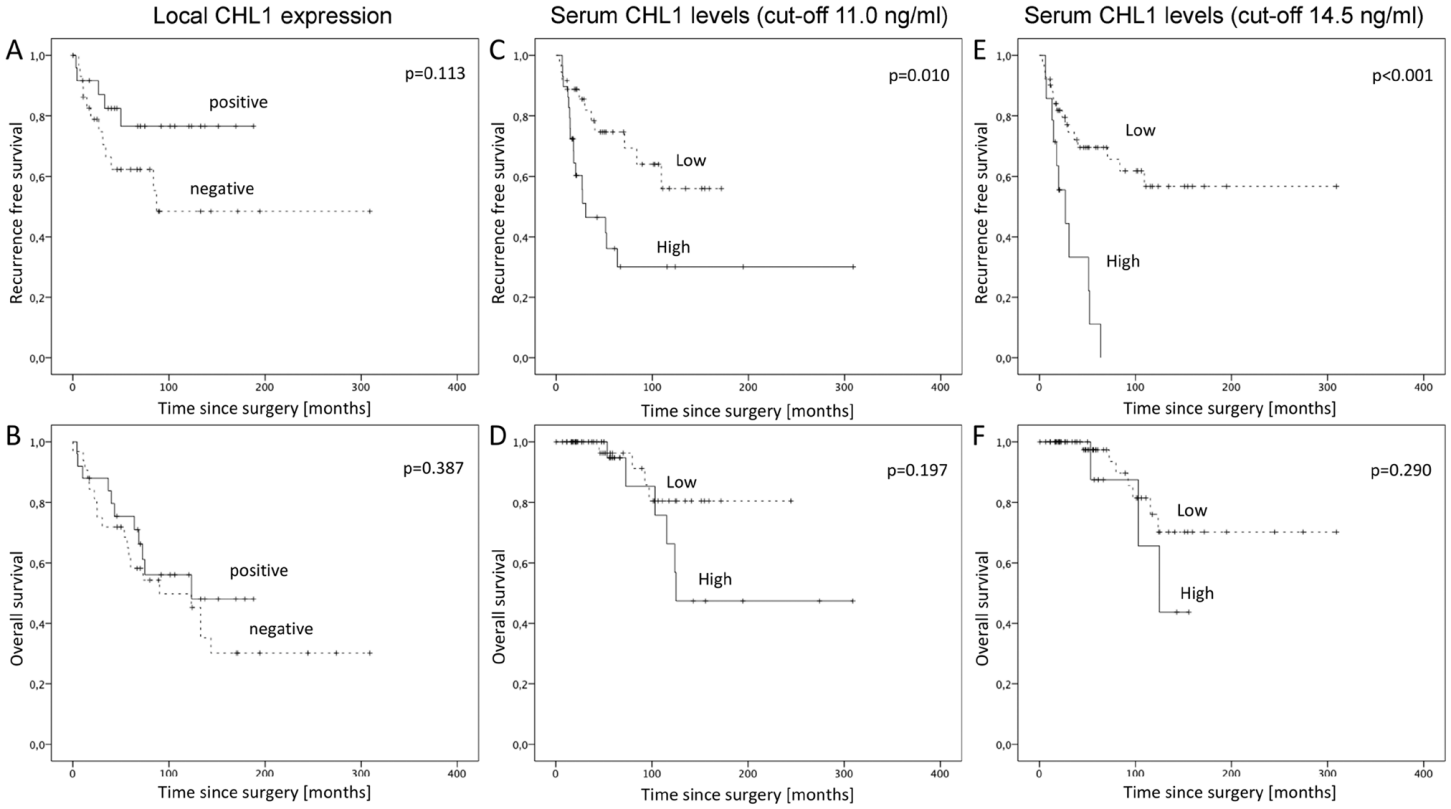
Kaplan-Meier survival analyses for recurrence free and overall survival in GIST. (**A**) Recurrence free survival for local CHL1 expression, (**B**) Overall survival for local CHL1 expression, (**C**) Recurrence free survival for serum CHL1 levels with cut-off values at 11.0 ng/ml, (**D**) Overall survival for serum CHL1 levels with cut-off values at 11.0 ng/ml, (**E**) Recurrence free survival for serum CHL1 levels with cut-off values at 14.5 ng/ml, (**F**) Overall survival for serum CHL1 levels with cut-off values at 14.5 ng/ml.

After confirmation of CHL1 expression on RNA as well as protein level in GIST we analyzed 58 samples of primary GIST on a TMA. The staining pattern of the CHL1 immunohistochemistry showed a predominantly membranous expression of the CHL1 molecule in GIST (Figure 2). Although some cytoplasmic staining was sometimes seen, this was always associated with a much higher staining level at the membranes. Primary tumors were CHL1-positive in 44.8% of cases (*n* = 26). Low tissue expression was significantly associated with advanced TNM stages (stage I and II versus III and IV, *p* = 0.036). In addition, a correlation with favorable tumor localization (gastric versus small and large intestine, esophagus (*p* = 0.039) was observed. Miettinen score failed to show a significant association with local CHL1 expression by a small margin (*p* = 0.078). When performing a Kaplan-Meier survival analysis, neither did recurrence free survival nor did overall survival reach significant values (*p* = 0.113 and *p* = 0.387, respectively). However, a trend towards a reduced recurrence free survival in GIST with decreased local expression was seen (Figure 3A and 3B). No further correlation between CHL1 protein expression levels and other clinicopathological parameters was found (Table 1).

**Table 1:** Association of clinicopathological characteristics of GIST patients with local CHL1 expression and serum CHL1 levels (cut-off 11.0 ng/ml)

		**local CHL1 expression**				**serum CHL1 level**			
		**total (*n* = 58)**	**negativ (*n* = 32)**	**positiv (*n* = 26)**	***p*-value **	**total (*n* = 81)**	**low (*n* = 41)**	**high (*n* = 40)**	***p*-value **
**Median age**, years (range)			59.9 (39–79)	63.7 (23–81)	0.169		62.3 (28–79)	56.5 (33–78)	0.056
**Sex**									
female		32	17 (53.1%)	15 (46.9%)		45	22 (48.9%)	23 (51.1%)	
male		26	15 (57.7%)	11 (42.3%)	0.468	36	19 (52.8%)	17 (47.2%)	0.451
**Disease Stage**									
T	1	8	4 (50.0%)	4 (50.0%)		7	6 (85.7%)	1 (14.3%)	
	2	22	12 (54.5%)	10 (45.5%)		21	15 (71.4%)	6 (28.6%)	
	3	11	6 (54.5%)	5 (45.5%)		20	10 (50.0%)	10 (50.0%)	
	4	14	8 (57.1%)	6 (42.9%)	0.991	18	7 (38.9%)	11 (61.1%)	0.073
	1 and 2	30	16 (53.3%)	14 (46.7%)		28	21 (75.0%)	7 (25.0%)	
	3 and 4	22	12 (54.5%)	10 (45.5%)	0.920	38	17 (44.7%)	21 (55.3%)	**0.023**
N	0	56	30 (53.6%)	26 (46.4%)		81	41 (50.6%)	40 (49.4%)	
	1	2	2 (100.0%)	0 (0.0%)	0.497	0	0 (0.0%)	0 (0.0%)	n/a
M	0	46	23 (50.0%)	23 (50.0%)		53	29 (54.7%)	24 (45.3%)	
	1	12	9 (75.0%)	3 (25.0%)	0.193	17	9 (52.9%)	8 (47.1%)	1.000
G	≤ 5/50 HPF	28	15 (53.6%)	13 (46.4%)		34	23 (67.6%)	11 (32.4%)	
	> 5/50 HPF	29	17 (58.6%)	12 (41.4%)	0.453	30	14 (46.7%)	16 (53.3%)	0.129
**UICC classification**									
I		24	12 (50.0%)	12 (50.0%)		19	16 (84.2%)	3 (15.8%)	
II		10	3 (30.0%)	7 (70.0%)		12	7 (58.3%)	5 (41.7%)	
IIIA		5	3 (60.0%)	2 (40.0%)		7	3 (42.9%)	4 (57.1%)	
IIIB		6	4 (66.7%)	2 (33.3%)		8	2 (25.0%)	6 (75.0%)	
IV		11	9 (81.8%)	2 (18.2%)	0.272	17	9 (52.9%)	8 (47.1%)	**0.044**
I and II		34	15 (44.1%)	19 (55.9%)		31	26 (83.9%)	5 (16.1%)	
III and IV		22	16 (72.7%)	6 (27.3%)	**0.036**	32	14 (43.8%)	18 (56.3%)	**0.021**
**Miettinen score**									
no		7	3 (42.9%)	4 (57.1%)		7	6 (85.7%)	1 (14.3%)	
very low		6	3 (50.0%)	3 (50.0%)		9	8 (88.9%)	1 (11.1%)	
low		12	8 (66.7%)	4 (33.3%)		9	4 (44.4%)	5 (55.6%)	
intermediate		10	2 (20.0%)	8 (80.0%)		9	6 (66.7%)	3 (33.3%)	
high		21	15 (71.4%)	6 (28.6%)	0.078	29	13 (44.8%)	16 (55.2%)	0.067
**Fletcher score**									
I°		6	2 (33.3%)	4 (66.7%)		6	5 (83.3%)	1 (16.7%)	
II°		11	7 (63.6%)	4 (36.4%)		11	9 (81.8%)	2 (18.2%)	
III°		10	6 (60.0%)	4 (40.0%)		11	6 (54.5%)	5 (45.5%)	
IV°		27	14 (51.9%)	13 (48.1%)	0.652	34	17 (50.0%)	17 (50.0%)	0.166
I° and II°		17	9 (52.9%)	8 (47.1%)		17	14 (82.4%)	3 (17.6%)	
III° and IV°		37	20 (54.1%)	17 (45.9%)	0.823	45	23 (51.1%)	22 (48.9%)	**0.041**
**Localization**									
stomach		41	17 (41.5%)	24 (58.5%)		45	38 (84.4%)	7 (15.6%)	
small bowell and others		17	15 (88.2%)	2 (11.8%)	**0.001**	25	17 (68.0%)	8 (32.0%)	0.098
**Recurrence**									
no		38	18 (47.4%)	20 (52.6%)		39	26 (66.7%)	13 (33.3%)	
yes		18	13 (72.2%)	5 (27.8%)	0.094	27	10 (37.0%)	17 (63.0%)	**0.024**

Significant values are highlighted in bold. n/a: not available; HPF: high power fields.

### Serum CHL1 levels are elevated in GIST patients indicating advanced tumor stages and reduced recurrence free survival

**Figure 4:**
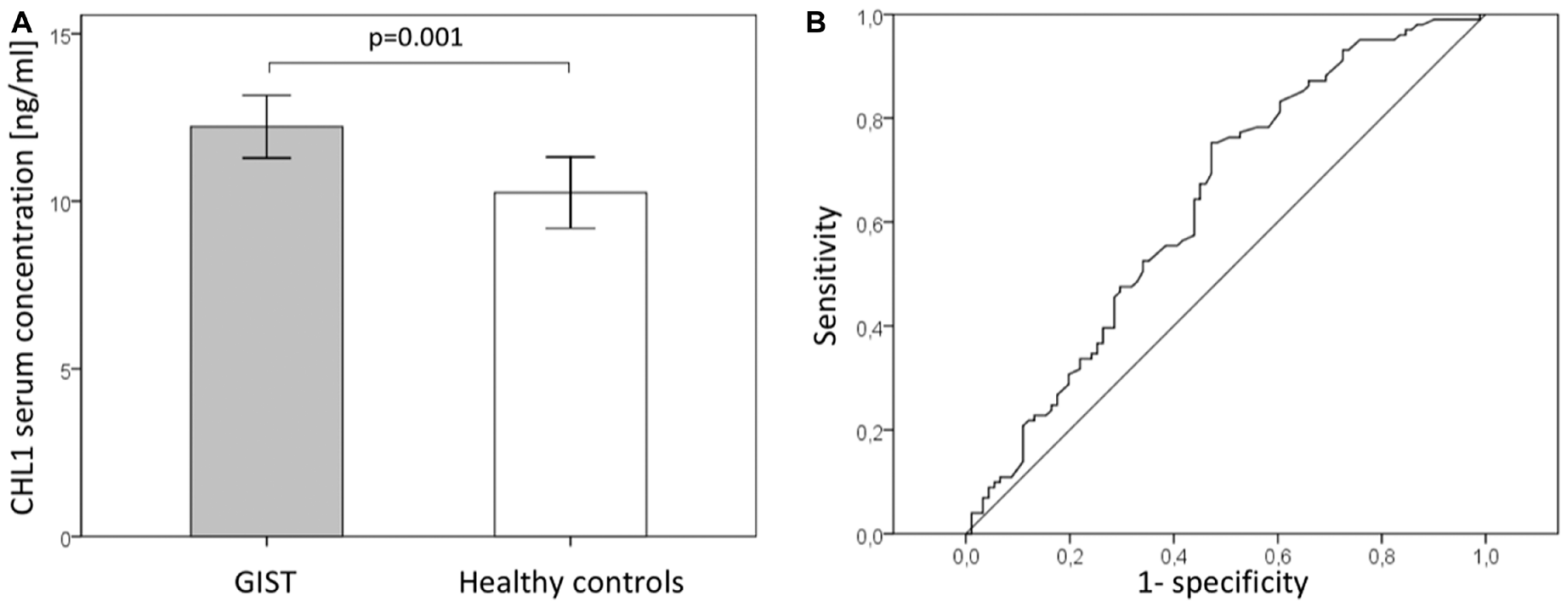
Serum CHL1 levels in GIST (**A**) Serum CHL1 levels of patients with GIST and healthy controls. (**B**) Receiver operating characteristic (ROC) curves of serum CHL1 levels for detecting GIST.

To further investigate the finding of predominantly soluble CHL1 fragments by Western Blot in human samples we analyzed the sera of 102 GIST patients by ELISA. A total of 91 sera of healthy volunteers were used as controls. Systemic CHL1 levels were significantly elevated in GIST patients (*n* = 102, median 11.6 ng/ml, standard deviation (SD) ±4.7 ng/ml) compared to healthy controls (*n* = 76, mean 8.5 ng/ml, SD ±5.1 ng/ml, *p* = 0.001; Figure 4A). A receiver operating characteristics curve was used to establish the sensitivity-specificity relationship for CHL1 levels (Figure 4B). The Youden index determined the cut-off level at 11.0 ng/ml (75th percentile at 14.5 ng/ml). For this cut-off a sensitivity of 72.3% and specificity of 52.7% with an area under the curve (AUC) of 0.690 was calculated. For the correlation of clinicopathological data with systemic CHL1 levels, patients were divided into a low-level (< 11.0 ng/ml) and a high-level serum CHL1 group (≥ 11.0 ng/ml). Cross tabulation showed a significant association of increased systemic CHL1 levels and advanced tumor sizes (pT3 and pT4 compared to pT1 and pT2; *p* = 0.023), higher UICC classification (I and II versus II and IV, *p* = 0.021), and increased Fletcher score (very low and low risk compared to intermediate and high risk; *p* = 0.041). Correlation with the Miettinen score missed significance by a small margin (*p* = 0.067). Other clinicopathological parameters did not show any significant differences. For further details see Table 1.

Survival curves plotted by the Kaplan-Meier analysis demonstrated a significant correlation of serum CHL1 levels and recurrence free survival (*p* = 0.010; Figure 3C), while overall survival did not show a significant association (*p* = 0.197; Figure 3D). Multivariate analysis failed to show an independent effect of systemic CHL1 levels for a cut-off at 11.0 ng/ml. When using the 75th percentile at 14.5 ng/ml as cut-off for systemic CHL1 levels, log rank analysis demonstrated an increase of the prognostic value for recurrence free survival (*p* < 0.001; Figure 3E). Overall survival still failed to reach significant parameters (*p* = 0.290; Figure 3F). Multivariate analysis further confirmed an independent prognostic effect of systemic CHL1 levels on recurrence free survival (*p* = 0.004) with a cut-off at 14.5 ng/ml. In addition, the mitotic count or grading of the GIST had a significant impact on recurrence free survival (*p* = 0.030). However, none of the other clinicopathological variables was of prognostic significance in multivariate analysis (Table 2).

**Table 2:** Prognostic value of serum CHL1 levels for recurrence free survival

	**univariate**			**multivariate (cut-off 11.0 ng/ml)**			**multivariate (cut-off 14.5 ng/ml)**		
	**HR**	**95% CI**	***p*-value **	**HR**	**95% CI**	***p*-value **	**HR**	**95% CI**	***p*-value **
**Sex**	0.913	0.522/1.595	0.748	2.910	1.095/7.737	**0.032**	2.086	0.665/6.544	0.208
**Age**	0.452	0.249/0.820	**0.009**	1.040	0.993/1.090	0.096	1.541	0.507/4.686	0.446
**Disease Stage**									
T	1.786	1.288/2.478	**0.001**	1.383	0.772/2.479	0.276	1.508	0.821/2.767	0.185
M	3.222	1.815/5.720	**0.001**	2.739	0.948/7.912	0.063	2.106	0.788/5.625	0.137
G	7.769	3.430/17.595	**0.001**	2.790	0.909/8.566	0.073	3.309	1.120/9.776	**0.030**
**serum CHL1 level**	0.439	0.154/1.249	0.123						
low vs high (cut-off 11.0 ng/ml)	2.633	1.222/5.673	**0.013**	1.640	0.592/4.541	0.341			
low vs high (cut-off 14.5 ng/ml)	4.098	1.826/9.196	**0.001**				4.610	1.620/13.123	**0.004**

Significant values are highlighted in bold. HR: hazard ratio; CI: confidence interval.

## DISCUSSION

Until now, clinicopathological parameters, as well as c-kit and PDGRFa mutation status, serve as prognostic markers in GIST. Diagnostic or serum markers with prognostic relevance for GISTs have not yet been reported. Hence, we wanted to investigate whether CHL1, which has been found in in other solid malignancies, is expressed by GISTs and might serve as potential diagnostic and prognostic marker in tissue and blood.

In this study, we were able to demonstrate that human GIST expresses CHL1 on mRNA as well as on protein level. We further demonstrated by immunohistochemistry, that the majority of CHL1 displayed a membranous localization and that 44.8% of the GIST yielded a significant local CHL1 expression. Reduced CHL1 tissue expression was associated with advanced tumor stages and a localization distant from the stomach indicating a worse tumor biology. In other entities, effects of CHL1 on biological functions of cancer cells have also been reported. A recent study demonstrated that inhibiting the CHL1 expression by microRNA-21 increases the invasiveness of tumor cells in colon adenocarcinomas [27]. Similar results have been shown for breast cancer cells in which CHL1 deficiency led to tumor formation, and a knockdown of CHL1 expression led to increased proliferation and invasion [21]. Of note, patient derived GIST predominantly showed soluble proteolytic CHL1 fragments with a molecular weight of 165 and 125 kDa in our study. Hence, our results implicate that the majority of the predominantly membranous CHL1 in human GISTs is cleaved. The ectodomain of CHL1 is a substrate of a disintegrin and metalloproteinases 8 (ADAM8) and beta-scretase 1 (BACE1) regulating cellular interactions [19, 20, 28]. Hence, one might hypothesize that cleavage of CHL1 along with other cell adhesion molecules leads to a functional downregulation that decreases cell adhesion. Soluble CHL1 or the remaining intracellular domain might then foster tumor cell migration and invasiveness. In accordance with this hypothesis, an association of decreased CHL1 expression with distant and lymph node metastases, as well as with reduced overall survival was reported for esophageal cancers [25]. The rate of CHL1 cleavage directly correlates with the amount of soluble CHL1 proteoforms, which are released into the environment and subsequently emitted into the blood stream. The possible role of mini-exon 8 in CHL1 isoform 2 has yet to be conclusively investigated. Interestingly, we were able to detect a significant higher amount of soluble CHL1 in the sera of GIST patients as compared to controls. For a cutoff at 11.0 ng/ml a good sensitivity (72.3%) and low specificity (52.7%) with an AUC of 0.690 was found indicating a sufficient detection of patients with GISTs. Other studies have investigated the role of anoctamin-1 (ANO1) positive circulating tumor cells as potential biomarker in GIST reporting a sensitivity of 64.2% and a specificity of 88.1% [29, 30]. The latter study also investigated the predictive role of systemic carcinoembryonic antigen (CEA) in GISTs describing a sensitivity of 69.5% and a specificity of 30.6% [30]. Hence, serum CHL1 might have a role in a panel of different serum markers in screening for GIST. Especially due to the non-invasiveness of obtaining a blood sample as opposed to taking a tissue biopsy, which might also be hampered by missing tumor tissue completely or by the collection of insufficient usable material, liquid biopsy studies must be further investigated. However, the low specificity of serum CHL1 would result in a high number of false positive results and further studies with larger cohorts must be performed to evaluate if it might have a diagnostic value alone, or in combination with other potential markers. In addition, we were able to link increased CHL1 serum levels in GIST patients with advanced tumors stages and high-risk tumors in accordance to the UICC classification and the Fletcher score. This is in line with a recent study by Kotani et al., describing a significant association between elevated CHL1’s blood-secretion and tumor size in a lung cancer xenograft mouse model [31]. To further support the prognostic importance of serum CHL1, GIST patients with high systemic CHL1 levels demonstrated a significant shorter recurrence free survival as compared to patients with low systemic CHL1 levels independent of other clinicopathological factors. In line with the latter finding, we found a trend towards earlier recurrences in patients with reduced local CHL1 expression. The divergent prognostic effect of local and systemic CHL1 levels seems to be a result of local CHL1 cleavage releasing CHL1 fragment into the blood stream. Interestingly, the CHL1 levels were not significantly increased in metastasized patients, leading to the hypothesis, that the CHL1 shedding might basically occurs in the primary tumor. However, no significant association of local CHL1 expression in univariate analysis with survival was found. In addition, systemic CHL1 levels (cut-off at 11.0 ng/ml) failed to reach an independent prognostic value in multivariate analysis. The latter results might be caused by the small cohort analyzed and further studies are needed to verify our data. Nevertheless, using the 75th percentile as cut-off for systemic CHL1 levels a clear independent prognostic effect was observed. Thus, the bad prognostic effect of increased serum CHL1 caused by increased local CHL1 cleavage might lead to a loosening of cell adhesions within the GIST intensifying the spread of tumor cells and thereby inducing earlier recurrences.

In conclusion, we demonstrated for the first time that GIST express CHL1 and that a significant amount of local CHL1 undergoes cleavage. Hence, reduced membranous CHL1 expression is associated with advanced tumor stages and unfavorable localizations of GIST. In addition, we were able to show that systemic CHL1 levels are increased in GIST patients and an inverse prognostic effect of local and systemic CHL1 levels was found. Finally, we were able to link systemic CHL1 levels with a shortened recurrence free survival independent of other clinicopathological parameters. Hence, serum CHL1 levels might have the potential to serve as a diagnostic and prognostic marker for GIST. Further studies with larger patient cohorts must validate the data of this preliminary study.

## MATERIALS AND METHODS

### Patients

The study was approved by the Medical Ethical Committee, Hamburg, Germany. Written informed consent was obtained from all patients before study inclusion. All procedures performed in this study involving human participants were in accordance with the ethical standards of the institutional and national research committee and with the 1964 Helsinki declaration and its later amendments or comparable ethical standards. Clinical data like sex, age at diagnosis, location and size of the tumor, Fletcher and Miettinen score, resection margin, metastasis, UICC stage according to the 8th edition, presence of recurrence as well as date and cause of death were obtained from a combination of clinical and pathological record review, reports of outside medical records and communication with patients and with their attending physicians. Overall survival was calculated from the date of surgery to the date of death or last follow-up. None of the patients received neo-adjuvant therapy.

### Tissue samples and tissue microarray

Fifty-eight patients with surgically resected GISTs, who were treated at the University Medical Center Hamburg-Eppendorf over a period of ten years were chosen retrospectively. All resected GISTs were diagnosed by immunohistochemical analyses (CD117, CH34, DOG1) and mutation analysis (KIT/PDGFRA) and interpreted as well as staged by two independent pathologists on a routine basis. Tissues were fixed in 4% buffered formalin and embedded in paraffin. Haematoxylin-eosin stained sections were cut from selected primary tumor blocks with representative tumor regions. Tissue cylinders with a diameter of 600 µm were punched out of the original donor block and arrayed on a new paraffin block using a semi-automated tissue arrayer. Subsequently, five µm sections of the complete TMA were constructed using the Paraffin Sectioning Aid System (Instrumentics, Hackensack, NJ, USA).

The patients were recruited after histological classification of the tumors including CD117 (c-kit; rabbit polyclonal) (Dako, Glostrup, Denmark), CD34 (mouse IgG1) (Novocastra Laboratories Ltd, Newcastle, United Kingdom), desmin (clone DE-R-11) (Dako), muscle actin (mouse IgG1; clone HHF35) (Enzo Diagnostics Inc., NY, USA), and S-100 protein (polyclonal) (Dako). The proliferative index was determined with Ki-67 (MIB1; IgG1) (Dako) and was categorized as mitotic count of < 5/50 high-power fields (HPF) and ≥ 5/50 HPF.

### Immunohistochemistry

The CHL1 staining protocol for paraffin tissues was optimized in an extensive multistep procedure on various benign and malignant tissues, modifying the staining protocol until selective staining with the lowest background signals were established [32, 33]. Freshly cut TMA sections were immunostained on one day and in one experiment. Slides were deparaffinized and exposed to heat-induced antigen retrieval for 5 minutes in an autoclave at 121°C in pH 7.8 Tris-EDTA-Citrate buffer. Primary antibody specific for CHL-1 (goat, polyclonal antibody; R&D Systems, USA; cat# AF2126; dilution 1:450) was applied at 37°C and pH 9.1 for 60 minutes. Bound antibody was then visualized using the EnVision Kit (Dako, Glostrup, Denmark) according to the manufacturer´s directions.

The staining intensity and the fraction of positive tumor cells were scored for each tissue spot, as described recently [34, 35]. Specimens were considered immunopositive for CHL1 if ≥ 30% of the tumor cells had clear evidence of immunostaining. Two independent investigators (EG and MT) performed the immunohistochemical analysis and scoring without knowledge of the patients’ identities or clinical statuses.

### Serum samples

For this study, 102 patients with GIST were chosen retrospectively. The serum samples were collected across Germany, Austria and Switzerland by the aid of a self-help organization for GIST patients called “Lebenshaus”. We selected patients on the basis of availability of specimens and did not stratify them due to rare occurrence and different treatment strategies. As control group, 91 blood bank donors were included in the study. Due to missing clinicopathological data of 21 patients a total of 81 patients were included into correlation and survival analyses.

### ELISA for the detection of soluble human CHL1

For the detection of soluble CHL1 (s-CHL1), 96-well flexible microtiter plates (Costar 9019) were coated with 50 µl per well of 10 µg/ml of capturing antibody (monoclonal murine IgG1 anti-human CHL1 antibody, Clone 316223, R&D Systems^®^; MN, USA) overnight. Wells were blocked with 3% w/v BSA Bovine serum albumin (BSA; Fraktion V, 98% purity, Sigma Aldrich, Munich, Germany) in PBS/T (PBS containing 0.05% v/v Tween) for 45 min and then incubated for 1 h with human sera diluted 1:5 in PBS. After five washes with PBS/T, bound protein was detected with biotin-conjugated goat mAb BAF2126 (anti-human CHL1 antibody, Lot Number URF01, R&D Systems^®^, MN, USA) followed by streptavidin-conjugated peroxidase using BMP as substrate. The color reaction was stopped by the addition of 10 mM H_2_SO_4_ and analyzed at 450 nm using an ELISA reader. Human CHL1–Fc protein (Catalog Number: 2126CH, R&D Systems^®^, MN, USA) served as an internal standard for the assay.

To ensure that the immunoassay was suitable for measuring clinical serum samples, reproducibility, linearity, and cross reactivity were examined. The assay showed negligible cross reactivity to L1, displayed excellent linearity with serial dilutions and showed < 10% coefficient of variation for intra- and inter-assay variability studies.

### CHL1 Western blots analysis

For analysis of CHL1 protein expression tissues were taken up in ice cold RIPA buffer (Pierce Biotechnology, Rockford, Il, USA) containing Halt Protease Inhibitor Mix (Pierce Biotechnology, Rockford, Il, USA) and lysed using a Dounce homogenisator. Lysates were centrifuged at 20,000 g at 4°C for 15 min and the supernatants were collected. Protein concentrations were estimated using the BCA Protein Assay (Pierce Biotechnology, Rockford, Il, USA). Lysates were then subjected to SDS-PAGE and Western blot analysis as previously described [36]. A polyclonal antibody raised against the extracellular domain of human CHL1 (CHL1/L1CAM-2 (AF2126; R&D Systems, USA) was used.

### Quantitative real time polymerase chain reaction (qPCR)

Total RNA from tissues was isolated using Trizol Reagent (Life Technologies). The collected upper phases were further processed with the RNeasy-Plus Kit (Qiagen, Hilden, Germany). High quality total RNA was used for cDNA synthesis using RT^2^ First Strand Kit (Qiagen, Hilden, Germany). PCRs were carried out in a LightCycler 480 II (Roche, Mannheim, Germany) using Maxima SYBR Green qPCR Master Mix (Thermo Scientific, VWR International – Germany GmbH, Darmstadt, Germany). The following primers have been used: panCHL1-F 5′-agaaaaggacagtcgcaatga-3′ (exon 7), iso1CHL1-F 5′-acagttaacagttcaaattccatca-3′ (exon boundaries 7/9), iso2CHL1-F 5′-caagttcatccacagaaattgg-3′ (exon 8), CHL1-R 5′-tggagttggcaagccttc-3′ (exon boundaries 9/10) and GAPDH 5′-CAG AAC ATC ATC CCT GCC TCT (sense), 5′-GCT TGA CAA AGT GGT CGT TGA G (antisense). The primers were designed to detect the total expression of CHL1 (panCHL1) by binding in exon 7 and at the exon boundary 9/10. The primers for the detection of CHL1 isoform 1 are at the exon boundaries 7/9 and 9/10. For the detection of the CHL1 isoform 2, the primers were placed on exon 8 and on the exon boundary 9/10. The following 3-step PCR program was used: initial denaturation 95°C for 10 min followed by 35 cycles 95°C for 15 s, 58°C for 20 s and 72°C for 20 s. Gene expression was normalized to GAPDH and relative expression was calculated using the ΔΔCt method.

### Statistical analysis

SPSS for Windows (Version 24, SPSS Inc., Chicago, IL USA) was used for statistical analysis. The cut-off level for serum CHL1 quantification was determined using the Youden index. Correlation were performed using a cross table and statistical analysis was done with Chi-Square test. Survival curves of the patients were plotted using the Kaplan-Meier method and analyzed using the log rank test. Median survival was not reached either for disease-free or overall survival. For that reason, survival data are presented as mean values. Cox regression analysis was used for univariate and multivariate analysis to assess the independent influence of CHL1 tissue expression or serum CHL1 levels and other covariates on recurrence and survival. *P* values less than 0.05 were considered statistically significant.

## Abbreviations

ADAM A: disintegrin and metalloproteinase
BACE1: Beta-secretase
CHL1: Neural cell adhesion molecule L1-like protein
CD: Cluster of differentiation
ELISA: Enzyme-linked immunosorbent assay
GIST: Gastrointestinal stroma tumors
HPF: High power fields
mRNA: Messenger ribonucleic acid
NrCam: Neuronal cell adhesion molecule
PDGRFA: Platelet-derived growth factor receptor A
P/S: Penicillin/streptomycin
RNA: Ribonucleic acid
RT-PCR: Real time polymerase chain reaction
TMA: Tissue microarray
TMN: TNM staging system
UICC: Union for International Cancer Control
qPCR: Quantitative real time polymerase chain reaction
